# Dual-Caspase-Mediated Apoptosis Underlies Peritoneal Cell-Free DNA Release After PD-Related Peritonitis

**DOI:** 10.3390/genes17040488

**Published:** 2026-04-19

**Authors:** Grazia Maria Virzì, Sabrina Milan Manani, Matteo Marcello, Angelo Porrovecchio, Claudio Ronco, Monica Zanella

**Affiliations:** 1 IRRIV—International Renal Research Institute Vicenza-Foundation, 36100 Vicenza, Italy; 2 Department of Nephrology, Dialysis and Transplant, San Bortolo Hospital, 36100 Vicenza, Italy

**Keywords:** cell free DNA, apoptosis, peritoneal dialysis

## Abstract

**Background/Objectives**: Cell-free DNA (cfDNA) is released into the circulation during inflammation-driven cellular injury and regulated cell death. Elevated cfDNA concentrations have been reported in several clinical settings, including chronic kidney disease, hemodialysis, and peritoneal dialysis (PD). We previously demonstrated that PD-related peritonitis induces an increase in circulating cfDNA; however, the mechanisms underlying cfDNA generation remained unclear. This study aimed (i) to confirm peritoneal cfDNA variation following peritonitis in PD patients, and (ii) to elucidate the apoptotic pathways responsible for cfDNA release. **Methods**: Fifty-four PD patients were enrolled and stratified into the following groups: Group A—no history of peritonitis (n = 25); Group B—remote peritonitis > 3 months prior (n = 21); Group C—recent peritonitis < 3 months prior (n = 8). cfDNA was quantified by qPCR. Apoptosis was assessed qualitatively by DNA laddering and quantitatively using ELISA assays for Caspase-3, Caspase-8 and Caspase-9. **Results**: cfDNA levels were significantly higher in patients with recent peritonitis compared to both other groups (*p* < 0.01). DNA laddering showed enhanced nucleosomal fragmentation, consistent with apoptosis. Caspase-3 concentrations were markedly increased in recent peritonitis (<3 months) and significantly correlated with cfDNA levels (ρ = 0.511, *p* < 0.01). Both Caspase-8 and Caspase-9 correlated with Caspase-3 (ρ = 0.57 and ρ = 0.47, respectively), indicating engagement of both extrinsic and intrinsic apoptotic pathways. **Conclusions**: In conclusion, peritoneal cfDNA in PD patients with peritonitis originates primarily from apoptosis and reflects dual-pathway caspase activation. cfDNA and Caspase-3 progressively decline with longer time elapsed from peritonitis, supporting their potential use as biomarkers for inflammatory activity and membrane recovery.

## 1. Introduction

Peritoneal dialysis (PD)-related peritonitis remains one of the most frequent and clinically relevant complications in PD patients. It accounts for a significant proportion of hospitalizations, technique failure, and mortality, with severe or recurrent episodes potentially inducing long-term sequelae such as encapsulating peritoneal sclerosis. Considerable center-to-center variability exists in peritonitis incidence, attributable to differences in patient selection, training, and infection-prevention practices [1,2]. Peritonitis induces both local and systemic inflammation that may persist beyond clinical recovery. Mesothelial cells orchestrate the inflammatory response within the peritoneal cavity, releasing cytokines and chemokines that propagate cellular stress [3,4]. In this inflammatory milieu, cells undergo regulated or catastrophic death, releasing fragments of nuclear DNA into circulation known as cell-free DNA (cfDNA).

cfDNA is typically double-stranded and composed of fragments smaller than genomic DNA (0.18–21 kb), detectable in numerous biological fluids under both physiological and pathological conditions. Its quantitative and qualitative profiling has become a versatile analytical tool, supporting applications ranging from non-invasive cancer diagnostics and minimal residual disease monitoring to prenatal screening, transplant rejection surveillance, and characterization of tissue-specific injury [5,6,7,8,9]. Within the peritoneal dialysis milieu, quantitative and qualitative interrogation of circulating cell-free nucleic acids has emerged as a biologically informative approach, as these analytes integrate signals from peritoneal membrane integrity, mesothelial and leukocyte turnover, sterile inflammation, and pathogen-driven immune activation. Their molecular profiling thus delineates a dynamic surrogate of local pathophysiological processes, with potential to enhance precision phenotyping and real-time monitoring of peritoneal membrane functionality [10].

Previous studies have reported elevated cfDNA in PD patients, with significantly higher concentrations after recent peritonitis. cfDNA levels decrease progressively with time from the acute event, suggesting active release during peritonitis and clearance once inflammation resolves. However, the molecular origin of cfDNA remains unclear: cfDNA may arise from apoptosis, necrosis, pyroptosis, or neutrophil extracellular traps (NETs), and its mechanistic characterization is essential for its clinical interpretation. While the association between cfDNA and apoptosis is well established, it does not exclude the contribution of other molecular mechanisms of cell death.

The objective of this study was to specifically investigate the apoptotic component underlying cfDNA generation in PD-related peritonitis. In particular, we aimed to elucidate the extent to which apoptosis contributes to cfDNA release by analyzing DNA fragmentation patterns and the activation of key apoptotic markers, including Caspase-3 (effector caspase), Caspase-8 (extrinsic pathway), and Caspase-9 (intrinsic mitochondrial pathway), in order to define the involvement of apoptotic cascades in this process.

## 2. Materials and Methods

### 2.1. Study Design and Patient Population

This observational case–control study was conducted in a cohort of adult patients undergoing peritoneal dialysis (PD) at San Bortolo Hospital in Vicenza. All patients were at least 18 years old and had been on PD therapy for a minimum of three months, ensuring that acute or transitional changes related to the initiation of dialysis did not influence the measurements. We specifically excluded individuals with active autoimmune diseases, malignancies, or acute infections unrelated to peritonitis, in order to avoid confounding elevations of cfDNA or apoptotic markers. Peritonitis episodes were defined using the diagnostic criteria established by the International Society for Peritoneal Dialysis (ISPD) [2], ensuring homogeneity and accuracy in patient classification. Based on clinical history and the timing of the most recent peritonitis episode, the population was divided into three groups:

1. Patients with no history of peritonitis (n = 25), serving as the baseline, inflammation-free reference group.
2. Patients with remote peritonitis, defined as an episode occurring more than three months prior to enrollment (n = 21).
3. Patients with recent peritonitis, defined as an episode occurring within the preceding three months (n = 8).

This distribution allowed us to capture the dynamic behavior of cfDNA and apoptotic markers across different stages of recovery from peritonitis.

The study protocol and informed consent procedure were approved by the Ethics Committee of San Bortolo Hospital (Deliberation No. 327, 08/2014). All participants provided written informed consent prior to inclusion.

### 2.2. Sample Collection and Peritoneal Preparation

Peritoneal samples were collected from each participant during routine clinic visits. The peritoneal effluent was then separated through a two-step centrifugation process: an initial spin at 3500 rpm for 10 min to remove intact cells, followed by a second, higher-speed centrifugation at 13,000 rpm for 10 min to eliminate cellular debris and ensure a clean, cell-free peritoneal fraction. This careful preparation was essential to avoid contamination from lysed leukocytes, which could artificially increase cfDNA measurements.

### 2.3. Extraction of Peritoneal cfDNA

cfDNA was extracted from peritoneal effluent samples using the MagPurix CFC DNA Extraction Kit (Zinexts Life Science Corp, New Taipei City, Taiwan). MagPurix is a fully automated nucleic acid extractor used for DNA extraction. It uses a disposable pre-loaded reagent cartridge based on the principle of separating nucleic acids from the remaining matrix using magnetic beads in solution. These are coated by a silica matrix with a high affinity for nucleic acids, which bind selectively. A magnetic field is then applied that holds the beads on the edge of the well, and after repeated washing, the DNA molecules subsequently eluted are obtained.

### 2.4. Quantification of Peritoneal cfDNA

cfDNA quantification was performed by real-time PCR using the Rotor-Gene 6000 system. For the quantification of total extracted cell-free DNA, a 137 bp fragment of the β-actin (ACTB) gene promoter was selected for amplification by real-time qPCR. The β-actin gene is commonly used as a housekeeping gene due to its stable and constitutive expression. Generally, these genes are described as being essential for basic cell survival, no matter what role they play in a specific tissue or organism. The primers used for this study were: forward 5′-3′: GCGCCGTTCCGAAAGTT; reverse 5′-3′: CGGCGGATCGGCAAA. The optimal amplification conditions were experimentally established using standard genomic DNA (Promega, Madison, WI, USA) on a Rotor-Gene 6000 instrument. Patient samples were analyzed in triplicate to ensure technical reproducibility. A volume of 10 µL of cfDNA was added to each reaction mixture (final volume of 15 μL). The real-time reaction mix consisted of 4.95 μL of deionized water, 1.25 μL of Eva Green (intercalating double strand (ds) DNA-binding dye) (Biotium, Hayward, CA, USA), 5 μL of magnesium-free real-time PCR buffer (TakaRa, Shiga, Japan), 1.5 μL of magnesium (TakaRa, Shiga, Japan), 0.5 μL of dNTP mixture (TakaRa, Shiga, Japan), 5 μM of each primer, and 0.3 μL of Takara Ex Taq R-PCR (TakaRa, Shiga, Japan). The real-time protocol of each amplification and each assay consisted of different steps, including an initial activation step at 95 °C for 3 min followed by 50 amplification cycles of 30 s denaturation, 30 s annealing at 65 °C (touchdown 1 °C for 10 cycles) and 30 s elongation, and a final elongation step at 72 °C for 5 min. The fluorescence acquisition was performed on the green channel. The real-time PCR products were heated to 99 °C and cooled for heteroduplex formation (the double-stranded molecule of DNA originated through the genetic recombination of single complementary strands), and melt was monitored by fluorescence emission through an appropriate denaturation range (50–99 °C). Melt-curve analysis, evaluating the dissociation characteristics of double-stranded DNA during heating, showed a single product-specific melting temperature with a mean of 93.5 °C for the β-actin gene. Each qPCR reaction was optimized using a standard curve generated from serial dilutions of genomic DNA with a known concentration (100 ng/μL; Promega). The standard dilution series included the following concentrations: 2 ng/μL, 200 pg/μL, 20 pg/μL, and 2 pg/μL. The amplification efficiency and reaction quality were evaluated based on the linear regression of the standard curves. Across all qPCR runs, the average coefficient of determination (R^2^) was 0.95, confirming a satisfactory level of linearity and reliability in the quantification process.

### 2.5. Assessment of Apoptosis

- Qualitative Evaluation: DNA Laddering

To explore whether cfDNA originated from apoptotic processes, we analyzed DNA fragmentation patterns using the Apoptotic DNA Ladder Extraction Kit (BioVision Abcam, Cambridge, UK). This method isolates low-molecular-weight DNA fragments characteristic of nucleosomal cleavage. Extracted DNA was separated on 1.5% agarose gels and stained with SybrSafe. Under ultraviolet light, apoptotic samples revealed a clear “ladder” pattern, consisting of bands spaced at approximately 180–200 base pair intervals. This qualitative approach provided a visual confirmation of apoptosis-driven DNA cleavage.

- Quantitative Analysis of Caspase

To complement the qualitative findings, we measured the peritoneal levels of several key enzymes involved in apoptosis:

- Caspase-3, the main executioner caspase responsible for DNA fragmentation;
- Caspase-8, which initiates the extrinsic (death-receptor-mediated) pathway;
- Caspase-9, which triggers the intrinsic (mitochondrial) pathway.

These enzymes were quantified using commercially available ELISA kits (eBioscience, San Diego, CA, USA). The assays rely on a colorimetric reaction proportional to enzyme concentration, read spectrophotometrically at 450 nm using a VICTORX4 Multilabel Plate Reader. All measurements were performed in triplicate to ensure accuracy and reproducibility. The combination of these markers enabled us to determine not only whether apoptosis was occurring but also which molecular pathways were involved.

### 2.6. Statistical Analysis

Statistical analyses were performed using SPSS version 15. Continuous variables were expressed as medians with interquartile ranges due to their non-normal distribution. Comparisons between two groups were performed using the Mann–Whitney U test, while the Kruskal–Wallis test was applied for three-group comparisons. Correlations between continuous variables—such as cfDNA and caspase levels—were evaluated using Spearman’s rho. A *p*-value below 0.05 was considered statistically significant. cfDNA concentrations were log-transformed prior to statistical analyses to improve distributional normality and better manage data and graphics.

## 3. Results

A total of 54 PD patients were included in this study (29 males and 25 females), with a mean age of 63.1 ± 16.3 years and a median PD vintage of 25.5 months (IQR: 13.2–49.4). In this cohort, the underlying causes of end-stage renal disease (ESRD) were diabetic nephropathy (25.9%), glomerulosclerosis (25.9%), nephroangiosclerosis (20.3%), autosomal dominant polycystic kidney disease (5.5%), vesicoureteral reflux (1.85%), pseudoxanthoma elasticum (1.85%), and unknown etiologies (18.5%). The three groups—patients with no history of peritonitis, patients with remote peritonitis (>3 months prior), and patients who had experienced peritonitis within the previous 3 months—showed similar demographic and clinical characteristics, with the exception of high-sensitivity C-reactive protein (hs-CRP). Hs-CRP levels were notably elevated among patients who had recently experienced peritonitis, indicating persistent systemic inflammation even after apparent clinical recovery (Table 1).

### 3.1. Peritoneal cfDNA Levels Across Patient Groups

**Figure 1:**
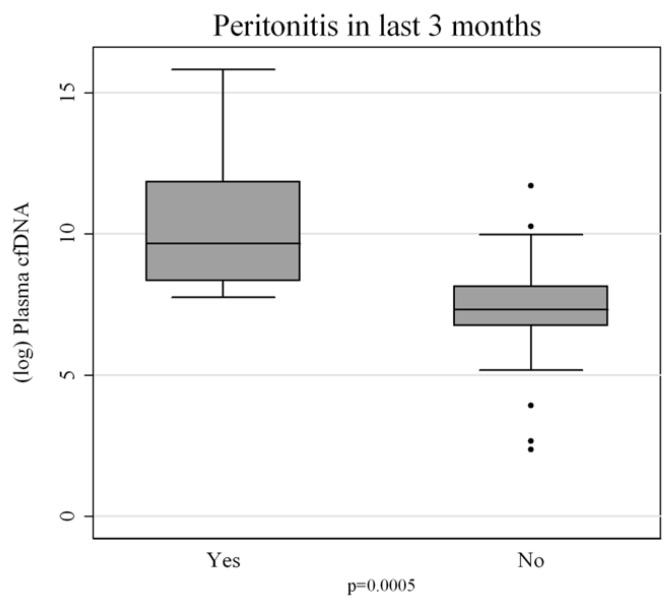
Comparison of circulating cell-free DNA (cfDNA, log scale) levels between peritoneal dialysis patients with and without a history of peritonitis. Patients with prior peritonitis (“Yes”) show significantly higher cfDNA levels compared to those without (“No”) (*p* = 0.0005). Box plots represent median, interquartile range, and outliers.

**Figure 2:**
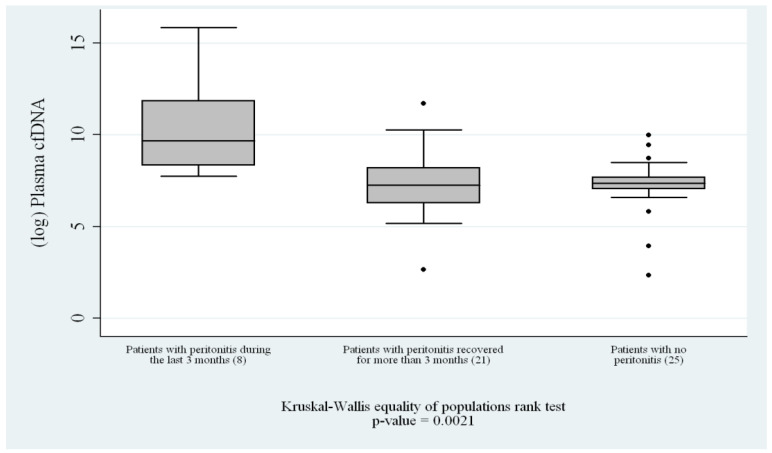
Comparisons between groups: patients with recent peritonitis (n = 8), patients with remote recent peritonitis who had recovered (n = 21), and patients with no history of peritonitis (n = 25) who had never experienced peritonitis. The *y*-axis shows log-transformed cfDNA levels. Overall comparison by Kruskal–Wallis, *p* = 0.0021.

The quantification of peritoneal cfDNA revealed a clear and progressive pattern associated with the timing of peritonitis. Patients who had never experienced peritonitis exhibited the lowest cfDNA levels, consistent with the absence of recent inflammatory or infectious stress (*p* = 0.0005) (Figure 1). Those with remote peritonitis showed intermediate cfDNA concentrations, suggesting that cfDNA levels gradually decline over time once the acute insult has resolved. The highest concentrations of cfDNA were found in patients with recent peritonitis (<3 months), with a statistically significant difference compared with both other groups (*p* < 0.01) (Figure 2). This pattern strongly suggests that cfDNA release is closely linked to the acute and subacute phases of peritonitis, and that the molecule may persist in circulation for weeks as a consequence of sustained or residual cellular turnover and inflammation.

### 3.2. Qualitative DNA Fragmentation: Evidence of Apoptosis

**Figure 3:**
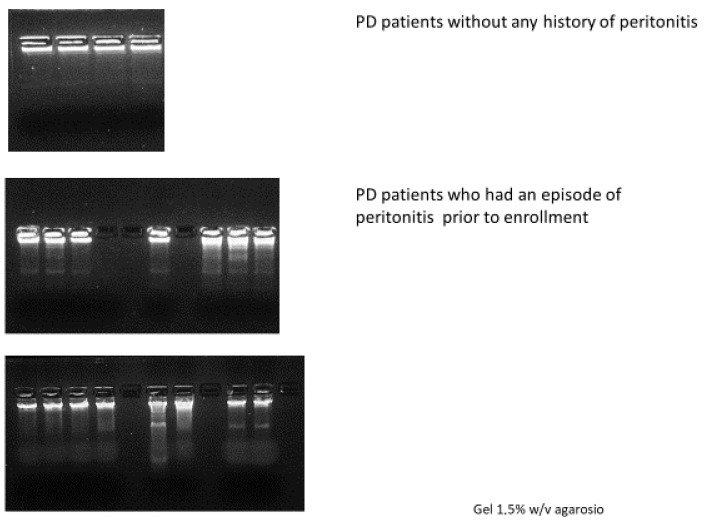
Qualitative assessment of DNA fragmentation by DNA ladder analysis on 1.5% (*w*/*v*) agarose gel in peritoneal dialysis (PD) patients. **Top panel**: patients without a history of peritonitis, showing predominantly intact DNA. **Middle panel**: patients with a prior episode of peritonitis before enrollment, displaying increased DNA fragmentation. **Bottom panel**: additional samples from patients with a history of peritonitis, exhibiting a pronounced “ladder” pattern consistent with apoptosis/eryptosis.

To explore the biological origin of cfDNA, we conducted a qualitative assessment of DNA fragmentation using DNA ladder analysis. This technique allowed us to visualize the characteristic nucleosomal cleavage pattern associated with apoptosis. The results aligned closely with cfDNA concentrations. Samples from patients without peritonitis history displayed minimal or absent fragmentation, consistent with physiological levels of cellular turnover. In contrast, samples from patients with remote peritonitis occasionally showed faint or partial laddering, suggesting that some degree of apoptotic activity may persist long after the acute episode. The most striking patterns were observed in patients who had recently recovered from peritonitis. These samples showed an intense and well-defined nucleosomal ladder, with DNA fragments corresponding to multiples of approximately 180–200 base pairs (Figure 3). This precise fragmentation pattern is a hallmark of apoptosis and clearly distinguishes it from necrosis, which produces longer and more heterogeneous DNA fragments. The presence of this laddering confirms that the increase in cfDNA is predominantly linked to apoptotic processes.

### 3.3. Caspase-3 Mirrors cfDNA Release

Caspase-3, the central effector caspase in the apoptotic cascade, was next evaluated to quantify apoptotic activity. The distribution of Caspase-3 levels closely paralleled both cfDNA concentrations and DNA laddering intensity.

**Figure 4:**
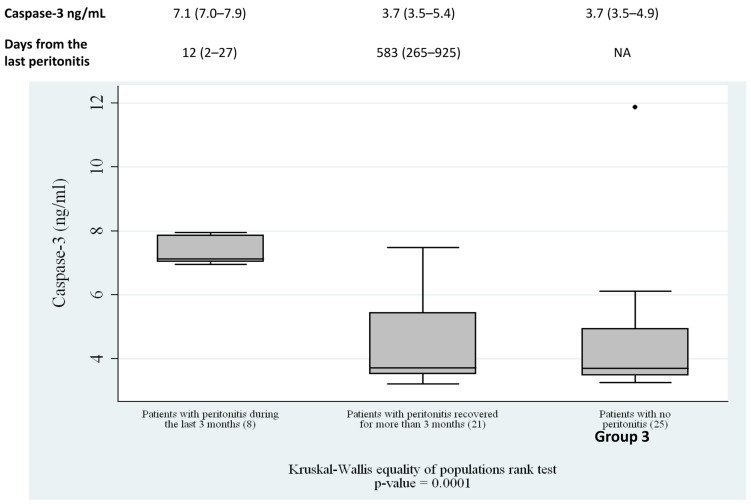
Comparisons of Caspase-3 between groups: patients with recent peritonitis (n = 8), patients with remote recent peritonitis who had recovered (n = 21), and patients with no history of peritonitis (n = 25) who had never experienced peritonitis (Kruskal–Wallis, *p* < 0.01). NA: not applicable.

Patients who had never experienced peritonitis exhibited low, baseline levels of Caspase-3, reflecting minimal systemic apoptotic activity. Those with remote peritonitis demonstrated slightly elevated but still moderate levels, compatible with a past inflammatory event that has since resolved. In contrast, patients with recent peritonitis showed a marked increase in Caspase-3, with median values approximately twice as high as those observed in the other groups (*p* < 0.01). Figure 4 reported Caspase-3 levels and days from the last peritonitis for each group (Figure 4). This robust increased level indicates that apoptosis is strongly stimulated during and immediately after peritonitis, and that Caspase-3 quantity is temporally aligned with the release of cfDNA into the bloodstream.

### 3.4. Correlation Between cfDNA and Caspase-3

**Figure 5:**
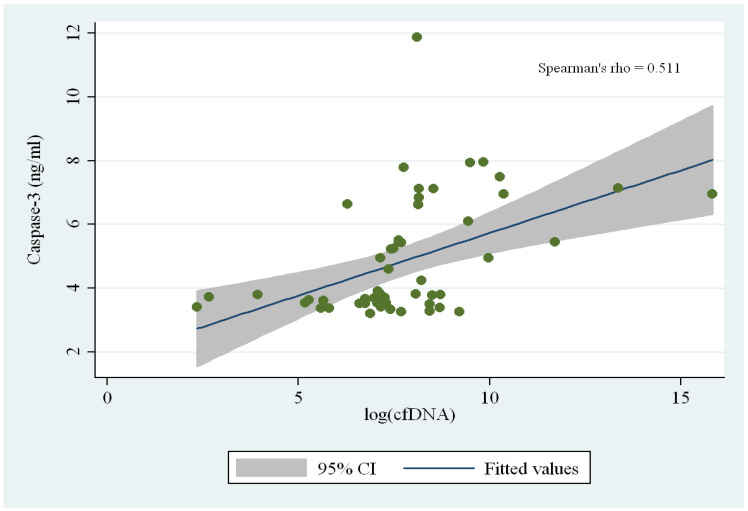
Correlation between Caspase-3 levels (ng/mL) and log-transformed cell-free DNA (cfDNA) in peritoneal dialysis patients. A moderate positive correlation was observed (Spearman’s ρ = 0.511), indicating that higher cfDNA levels are associated with increased caspase-3. The solid line represents the fitted regression line, with the shaded area indicating the 95% confidence interval.

A strong and significant correlation emerged between cfDNA concentrations and Caspase-3 levels (Spearman’s ρ = 0.511, *p* < 0.01) (Figure 5). This association reinforces the hypothesis that apoptosis, rather than necrosis or other forms of cell death, is the predominant source of peritoneal cfDNA during peritonitis. As Caspase-3 mediates the cleavage of nuclear DNA during programmed cell death, higher levels of cfDNA reflect the increased number of apoptotic events occurring in the aftermath of the inflammatory insult.

### 3.5. Activation of Extrinsic and Intrinsic Apoptotic Pathways

To further elucidate the mechanisms underlying apoptosis, we quantified Caspase-8 and Caspase-9—key initiator caspases of the extrinsic (death-receptor-mediated) and intrinsic (mitochondrial) pathways, respectively. Both caspases were detectable across subjects and showed a clear relationship with Caspase-3 levels. Caspase-8 and Caspase-9 correlated significantly with Caspase-3 (ρ = 0.57 and ρ = 0.47, respectively), indicating that peritonitis leads to the simultaneous activation of both apoptotic pathways (both caspases increased levels) (Table 2). This dual activation suggests that inflammatory mediators (e.g., TNF-α) and mitochondrial stress coexist during peritonitis and jointly contribute to widespread apoptotic signaling. The convergence of these pathways on Caspase-3 provides a mechanistic explanation for the pronounced DNA fragmentation and cfDNA release observed in recent peritonitis.

## 4. Discussion

Peritoneal-dialysis-related peritonitis represents a complex inflammatory insult that affects not only the peritoneal cavity but also the systemic compartment. The present study provides an integrated and coherent picture of how this inflammatory event translates into measurable biological signals in the bloodstream, particularly through the release of cell-free DNA (cfDNA) and the activation of apoptosis-related caspases. Our results confirm and expand previous findings by demonstrating that cfDNA levels rise significantly in the months following an episode of peritonitis and that this increase is tightly associated with activation of apoptotic pathways [10,11,12,13,14,15,16]. In this context, it is important to underline that the present study was deliberately designed to specifically investigate the apoptotic contribution to cfDNA generation. Accordingly, our analyses were focused on established markers of apoptosis, allowing us to define its role in this setting. However, this targeted approach does not exclude the possible involvement of additional cell death mechanisms, such as pyroptosis, necrosis, or neutrophil extracellular trap (NET) formation, which may also contribute to cfDNA release in the highly inflammatory milieu of PD-related peritonitis.

A key element emerging from our data is that patients with recent peritonitis (<3 months) exhibit the highest concentrations of cfDNA. This observation aligns with the clinical understanding of peritonitis as a condition capable of generating sustained systemic inflammation, even when the acute symptoms appear to have subsided. The elevation of hs-CRP we observed in this subgroup supports the notion that inflammatory activity remains heightened well after the apparent clinical resolution of the episode. In this inflammatory context, cfDNA likely reflects the cumulative effect of cellular stress, immune activation, and apoptotic turnover of mesothelial and inflammatory cells. Our data are consistent with the work of Lam, who demonstrated that PD patients continue to exhibit prolonged systemic inflammation even one month after apparent clinical remission of peritonitis [17].

The qualitative analysis of peritoneal DNA fragments via DNA laddering added an important mechanistic layer to these findings. The markedly visible nucleosomal ladder in patients with recent peritonitis is characteristic of apoptosis, distinguishing this form of cell death from necrosis, which typically produces longer, irregular DNA fragments. The presence of these distinct fragments suggests that peritonitis stimulates a regulated form of cellular dismantling, in which endonucleases cleave chromatin into well-defined units [18,19].

This apoptotic signature reinforces the idea that the increase in cfDNA is not merely a by-product of tissue damage but rather a biological consequence of a programmed cell death process activated in response to inflammatory stimuli.

The central role of apoptosis was further supported by the clear rise in Caspase-3 levels in patients with recent peritonitis. Caspase-3 acts as a final executioner in the apoptotic cascade, integrating signals from both the extrinsic and intrinsic pathways. Its strong correlation with cfDNA suggests that cfDNA is generated predominantly at the moment when Caspase-3 becomes activated, and downstream effectors initiate DNA fragmentation. The decline in Caspase-3 levels with increasing time from peritonitis mirrors the progressive reduction in cfDNA, suggesting a dynamic process in which apoptosis is most intense near the acute phase and gradually diminishes as the inflammatory environment resolves.

The involvement of both Caspase-8 and Caspase-9 adds depth to this biological narrative. Their correlation with Caspase-3 indicates that both apoptotic pathways—extrinsic, mediated by cell-surface receptors, and intrinsic, triggered by mitochondrial stress—contribute to the apoptotic burden observed in peritonitis. This dual activation is physiologically plausible: inflammatory cytokines such as TNF-α can trigger the extrinsic pathway, while oxidative stress and mitochondrial damage, both well-described consequences of peritoneal inflammation, can initiate the intrinsic one. Together, these pathways converge on Caspase-3, amplifying apoptotic signaling and enhancing cfDNA release.

These findings fit well with the existing literature on apoptosis-driven cfDNA production in other inflammatory conditions. The strong association between cfDNA and apoptotic events has also been highlighted by Moreira et al., who analyzed cfDNA release following apoptosis in a cohort of febrile patients, concluding that cfDNA could serve as a direct marker of cellular apoptosis [20]. Similarly, Clementi et al. reached the same conclusion in a population of critically ill patients with sepsis [21].

What differentiates the current study is the ability to observe this dynamic specifically in the context of peritoneal dialysis, where cfDNA may not only reflect systemic inflammation but also provide insight into the status of the peritoneal membrane. The observed decline in cfDNA over time suggests that cfDNA could eventually serve as a marker to monitor recovery after peritonitis, complementing clinical assessment and traditional laboratory parameters.

From a clinical perspective, our results highlight the potential of cfDNA as an informative biomarker in PD patients. While cfDNA measurement is already gaining traction in oncology and critical-care settings, its application in nephrology—and specifically in PD—remains underexplored. The tight link between apoptosis and cfDNA observed here suggests that cfDNA may reflect membrane stress, ongoing inflammation, or residual cellular injury, offering clinicians a non-invasive window into processes otherwise difficult to monitor. Future studies could evaluate whether cfDNA patterns predict recurrent peritonitis, technique failure, or long-term membrane alterations.

The study has limitations, including the modest size of the recent peritonitis subgroup and its single-center design. In addition, our analysis was specifically focused on apoptotic pathways and therefore does not capture the full spectrum of cell death mechanisms potentially contributing to cfDNA release. In particular, we acknowledge that pyroptosis—mediated by inflammasome activation and Caspase-1 signaling—may also play a relevant role in this inflammatory setting. The absence of pyroptosis-specific markers (e.g., Caspase-1, IL-1β, and gasdermin D) thus represents an additional limitation of our study. Furthermore, the lack of comprehensive inflammatory profiling, including the quantification of cytokines and other inflammatory indices, limits our ability to fully characterize the inflammatory milieu associated with cfDNA release.

Nonetheless, the consistency across cfDNA, DNA laddering, and multi-caspase analysis strengthens the reliability of our findings. Expanding this research with future studies with longitudinal sampling and integrating cytokine profiling, pyroptosis-related markers, and NETosis indicators will be essential to better define the relative contribution of different forms of cell death to cfDNA release in PD-related peritonitis.

In conclusion, our study provides robust evidence that cfDNA in PD-related peritonitis originates predominantly from apoptosis and that both intrinsic and extrinsic pathways are activated during the inflammatory insult. This mechanistic insight supports the role of cfDNA as a potential biomarker for monitoring peritonitis activity and recovery in PD patients. Unfortunately, the transition of cfDNA analysis from a research tool to a routine clinical assay in PD is still gradual, primarily due to pre-analytical challenges such as sample handling, standardization of isolation methods, and fragment quantification. Overcoming these hurdles will be essential to fully exploit cfDNA as a reliable biomarker for assessing peritonitis onset, severity, and resolution, ultimately enabling more precise and timely management of PD patients.

## Figures and Tables

**Table 1:** Characteristics of PD patients.

	25 pts Without Peritonitis	21 pts with Peritonitis Recovered	8 pts with Peritonitis in the Last 3 Months	*p*-Value
Sex (Male/Female)	14M/11F	11M/10F	4M/4F	NS
Age (years)	63.0 ± 16.3	63.6 ± 16.5	63.5 ± 16.7	NS
Time on PD (months)	26.4 (13.3–51.4)	23.6 (12.8–49.3)	25.1 (16.3–39.1)	NS
Type of PD (APD/CAPD)	8 APD/17 CAPD	7 APD/14 CAPD	2 APD/6 CAPD	NS
Weekly Creatinine Clearance (l/sett/1.73 m^2^)	72.7 (53.0–102.6)	72.7 (52.1–106.1)	87.7 (61.8–108.6)	NS
Weekly Kt/Vurea	1.95 (1.63–2.21)	1.94 (1.63–2.18)	2.02 (1.63–2.21)	NS
Hemoglobin (g/dL)	11.9 (11.4–12.9)	11.9 (11.5–12.9)	11.6 (11.1–12.9)	NS
HS CRP (mg/dL)	0.33 (0.29–0.82)	0.30 (0.29–0.82)	0.68 (0.30–2.85)	0.012
Albumin (g/dL)	3.0 (2.7–3.2)	3.0 (2.8–3.2)	3.0 (2.8–3.1)	NS
Diuresis (mL)	600 (25–1075)	600 (147–1175)	650 (54–1094)	NS

NS: not significant.

**Table 2:** Correlations between Caspase-3, Caspase -8 and Caspase-9.

	Spearman’s Rho	*p*
Caspase-3/Caspase-8	0.57	0.001
Caspase-3/Caspase-9	0.47	0.001

## Data Availability

The data presented in this study are available on request from the corresponding author.
